# Pubic disjunction following vaginal delivery in a multiparous woman: A case report

**DOI:** 10.1016/j.amsu.2021.102629

**Published:** 2021-07-27

**Authors:** Telmoudi Ely Cheikh, Elmiski Fatiha, Ikouch Khadija, Charquaoui Malak, Jalal Mohamed, Lamrissi Amine, Fichtali Karima, Bouhya Said

**Affiliations:** Department of Gynecology and Obstetrics, Faculty of Medicine and Pharmacy, Hassan II University, IBN ROCHD University Hospital, Casablanca, Morocco

**Keywords:** Single-fetal pregnancy, Multiparity, Vaginal delivery, Pelvic pain, Pubic separation

## Abstract

**Introduction:**

Pubic disjunction syndrome has been identified as responsible for pain during pregnancy and post partum; it is favored by twins and traumatic events such as extraction maneuvers.

**Case report:**

A 38-year-old woman, 5G/5P, with no pathological history, who presented 2 days postpartum with no notion of instrumental extraction, with severe pelvic symphysis pain with functional impotence. Examination revealed elective pain on palpation of the pubic symphysis without radiation. X-ray of the pelvis showed a 15-mm enlargement of the symphysis. CT scan of the pelvis showed a 17-mm disjunction of the pubic symphysis. The patient received analgesic treatment with pelvic bandage and anticoagulant therapy with good clinical response.

**Discussion:**

Peripartum pubic disjunction is a rare entity whose etiologies are still poorly understood, although multiparity, fetal macrosomia, extraction maneuvers, and joint pathologies have been incriminated. Symptomatology includes pubic symphysis pain. Conventional radiology shows an abnormal space at the inter-symphysial joint greater than 10 mm. Medical treatment with pelvic bandage, rest, physical therapy and physiotherapy allows a favorable evolution, otherwise infiltrations containing a local anesthetic and corticosteroids. Surgery is indicated if the diastasis is greater than 4 cm.

**Conclusion:**

Symphyseal disjunction is evoked in front of any pelvic pain in peripartum, the diagnosis is made at the radiology of the pelvis with intersymphysis space higher than 10 mm. The treatment is medical associating an analgesia or a local infiltration, a rest and a physiotherapy. Pelvic bandaging and surgical treatment are reserved for complicated forms.

## Highlights

• Suspected diagnosis in front of any symphyseal pubic hyperalgesia syndrome of peripartum.
• Diagnosis confirmed by radiological examinations.
• Treatment is essentially symptomatic.
• Recourse to surgical treatment in case of significant disjunction.

## 1 Introduction

The pubic disjunction syndrome is identified as responsible for pain during pregnancy and postpartum; it is favored by twins and traumatic events such as extraction maneuvers [1,2]. The management of hyperalgesic patients concerned by this pathology must be rapid and adapted in order to ensure autonomy and comfort for the parturient. We report here the case of a patient who presented a hyperalgesic pubic disjunction in the aftermath of a single fetal pregnancy delivered by vaginal delivery in a multiparous woman. This work has been reported with respect to the SCARE 2020 criteria [3].

## 2 Case report

**Fig. 1:**
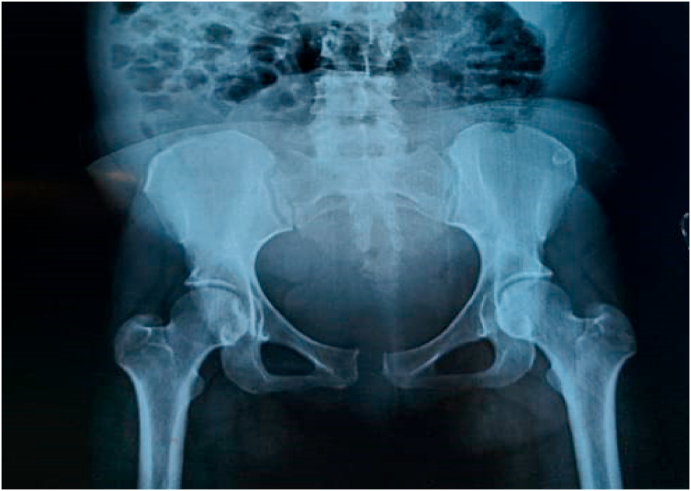
The X-ray of the pelvis showed an enlargement of the pubic symphysis evaluated at 15 mm.

**Fig. 2:**
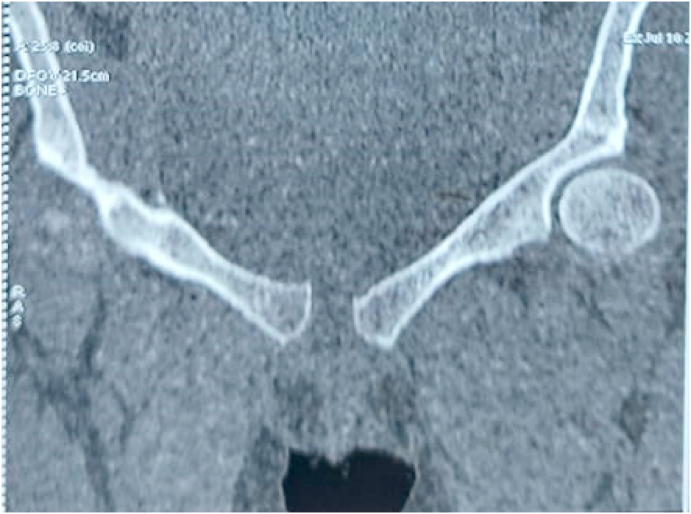
Evidence of a disjunction of the pubic symphysis measured at 17 mm with infiltration of the adjacent soft tissues.

A 38 year old female patient, 5G/5P, without any particular pathological history, presented 2 days post partum after a vaginal delivery of a mono fetal pregnancy without any notion of trauma or instrumental extraction for an intense pain of the pelvic symphysis without irradiation with functional impotence making walking impossible, imposing a strict dorsal decubitus. She was admitted to our structure in an ambulance accompanied by a nurse. The clinical examination revealed an elective pain on palpation of the pubic symphysis, reproduced on palpation of the iliac wings, without radiation. X-ray of the pelvis showed enlargement of the pubic symphysis assessed at 15 mm with significant local remodeling (Fig. 1). The CT scan of the pelvis showed a disjunction of the pubic symphysis evaluated at 17 mm (Fig. 2). The oral analgesia then put in place combined paracetamol (Doliprane 1 g/6 h), a ketoprofen type NSAID (Biprofenid 150 mg/12 h), and nefopam (Acupan 20 mg/6 h), with pelvic bandaging and anticoagulant treatment with a good clinical response during her hospitalization. Patient declared discharged after 5 days of hospitalization with good clinical improvement. During her one month follow-up the patient presented no pain.

## 3 Discussion

The incidence of pubic disjunction syndrome in the peripartum is estimated between 1/300 and 1/30,000 in the literature [4,5], in fact 22 % of parturients may have pain at the level of the pubic symphysis, this pain is excruciating in 5–8% of parturients and 7 % of parturients have this symptomatology in the post partum period [6,7].

The etiologies of this syndrome are still poorly understood, although some authors incriminate multiparity, fetal macrosomia, extraction maneuvers, joint pathologies and trauma of the pubic joint [8,9]. In the clinical case reported, the patient had multiparity as a risk factor. To date, there is only one case in the literature of postpartum pubic disjunction in the absence of any risk factor [10].

The typical symptomatology seems to involve pubic symphysis pain with inguinal radiations associated with sacroiliac joint pain [11]. Some even describe edema of the symphysis and true palpation of an intersymphysis space [12]. The intensity of the pain is described as variable but always exacerbated by movements involving the symphyseal joint, such as standing, walking, climbing stairs, or carrying heavy loads [11]. The paraclinical diagnosis is based on conventional radiology. Indeed, an X-ray of the pelvis from the front may reveal an abnormal space at the level of the intersphysial joint, bearing in mind that a space of less than 9 mm is physiological and that a space of more than 10 mm is considered pathological [9]. However, the degree of separation observed does not appear to be correlated with the severity of the symptoms [8], and a pubic disjunction symptomatology may be observed in the absence of radiological signs [13]. The differential diagnoses to be evoked in the face of pubic pain in pregnancy and/or postpartum are all osteoarticular pathologies, such as rheumatoid arthritis, ankylosing spondylitis, and multiple myeloma. Traumatic pathologies should also be considered, such as fractures and traumatic separation of the symphysis pubis [11]. Medical treatment combining pelvic bandaging, rest, physiotherapy and physical therapy has led to a favorable evolution, otherwise infiltrations with local anesthetics and corticoids can be used [14]. In case of immobilization, anticoagulant treatment must be associated even for simple disjunctions. According to the literature, surgical treatment with reduction under general anaesthesia may even be indicated in case of diastasis greater than 4 cm [15].

## 4 Conclusion

Symphyseal disjunction syndrome must be evoked in front of any pelvic pain of pregnancy and postpartum, the diagnosis is easily made thanks to a radiology of the pelvis showing an intersphysial space higher than 10 mm. The initial early management is medical with a combination of oral analgesia or local infiltration, rest and physical therapy. Pelvic bandaging and surgical treatment are reserved for cases of significant diastasis and must be associated with preventive anticoagulation in case of immobilization.

## Sources of funding

None.

## Ethical approval

I declare on my honor that the ethical approval has been exempted by my establishment.

## Author contribution

TELMOUDI ELY CHEIKH: Corresponding author writing the paper. El MISKI FATIHA: writing the paper. IKOUCH KHADIJA: writing the paper. CHARQAOUI MALAK: writing the paper. JALAL MOHAMED: study concept. LAMRISSI AMINE: study concept. FICHTALI KARIMA: study concept. BOUHYA SAID: correction of the paper.

## Trial registry number

None.

## Guarantor

DR TELMOUDI ELY CHEIKH.

## Consent

Written informed consent was obtained from the patient for publication of this case report and accompanying images. A copy of the written consent is available for review by the Editor-in-Chief of this journal on request.

## Provenance and peer review

Not commissioned, externally peer-reviewed.

## Declaration of competing interest

The authors report no declarations of interest.
